# Artificial intelligence: clinical applications and future advancement in gastrointestinal cancers

**DOI:** 10.3389/frai.2024.1446693

**Published:** 2024-12-20

**Authors:** Abolfazl Akbari, Maryam Adabi, Mohsen Masoodi, Abolfazl Namazi, Fatemeh Mansouri, Seidamir Pasha Tabaeian, Zahra Shokati Eshkiki

**Affiliations:** ^1^Colorectal Research Center, Iran University of Medical Sciences, Tehran, Iran; ^2^Infectious Ophthalmologic Research Center, Ahvaz Jundishapur University of Medical Sciences, Ahvaz, Iran; ^3^Department of Internal Medicine, School of Medicine, Iran University of Medical Sciences, Tehran, Iran; ^4^Department of Microbiology, Faculty of Sciences, Qom Branch, Islamic Azad University, Qom, Iran; ^5^Alimentary Tract Research Center, Clinical Sciences Research Institute, Imam Khomeini Hospital, Ahvaz Jundishapur University of Medical Sciences, Ahvaz, Iran

**Keywords:** artificial intelligence, gastrointestinal cancers, machine learning, deep learning, early detection, diagnosis, treatment response, survival prediction

## Abstract

One of the foremost causes of global healthcare burden is cancer of the gastrointestinal tract. The medical records, lab results, radiographs, endoscopic images, tissue samples, and medical histories of patients with gastrointestinal malignancies provide an enormous amount of medical data. There are encouraging signs that the advent of artificial intelligence could enhance the treatment of gastrointestinal issues with this data. Deep learning algorithms can swiftly and effectively analyze unstructured, high-dimensional data, including texts, images, and waveforms, while advanced machine learning approaches could reveal new insights into disease risk factors and phenotypes. In summary, artificial intelligence has the potential to revolutionize various features of gastrointestinal cancer care, such as early detection, diagnosis, therapy, and prognosis. This paper highlights some of the many potential applications of artificial intelligence in this domain. Additionally, we discuss the present state of the discipline and its potential future developments.

## 1 Introduction

Gastrointestinal (GI) cancers, as a group of malignancies that affect the digestive system, include the esophagus, stomach, liver, pancreas, and colorectum. These malignancies are prevalent and highly lethal on a global scale. With the presence of several sites prone to malignant transformation and continuous exposure to carcinogens, GI cancers constitute 26% of the total global cancer cases and contribute to 35% of the worldwide mortality from cancer (Wong et al., 2022). The incidence of esophageal, gastric, and liver cancers had higher prevalence rates in Asia, but colorectal and pancreatic cancers were shown to be more prevalent in Europe and North America (Arnold et al., 2020). The prevalence and mortality rates of age-adjusted non-cardia gastric cancer (NCGC) have demonstrated a downward trend, but the occurrence of esophageal adenocarcinoma, as well as malignancies affecting the cardia gastric, colorectal, liver, and pancreas, have exhibited an upward trajectory among those under the age of 50 over the past 25 years (Laszkowska et al., 2020). Various diagnostic procedures are developed for the identification of distinct forms of GI malignancies. Several commonly used techniques for diagnosing medical conditions include endoscopy, biopsy, imaging modalities (such as ultrasound), computed tomography (CT) scan, magnetic resonance imaging (MRI) scan, blood tests (such as tumor markers), stool testing (such as fecal occult blood test), and genetic tests (such as gene mutations or microsatellite instability) (Xie et al., 2022). Early detection, precise diagnosis, efficient therapy, and accurate treatment monitoring can improve the prognosis and survival rates of patients with GI malignancies. Screening programs (e.g., regular screenings of colon cancer) have significantly contributed to the timely detection of individuals at risk for certain malignancies (Lin et al., 2021). Nevertheless, several GI malignancies lack efficient screening methods and deal with significant challenges in early detection (Del Chiaro et al., 2014). In addition, the diagnosis of GI cancers typically involves invasive procedures, such as biopsy and subsequent pathological investigation after surgical resection. Many GI malignancies lack reliable biomarkers, even after diagnosis, that could serve as definitive tools for staging and prognosis to support clinical decision-making (Wesdorp et al., 2021).

As medical imaging technology evolves, so has picture interpretation, particularly computer-assisted analyses. Philippe Lambin invented radiomics in 2012, which uses high-throughput data to analyze medical pictures (Lambin et al., 2012). The program helps diagnose, classify, and categorize malignancies, as well as predict outcomes using several endpoints (Lambin et al., 2012). Oncology is increasingly using AI to detect, diagnose, predict therapeutic response, and predict GI cancer survival (Huang C.-M. et al., 2020; Qiu et al., 2022; Wang Z. et al., 2023). Artificial Intelligence (AI) is the application of technology to construct robots and computers that can mimic human cognitive capabilities, including decision-making, data analysis, and language translation. AI covers several associated but different cancer subfields, including machine learning (ML) and deep learning (DL) (Adlung et al., 2021; Sánchez-Martínez et al., 2019; Dias and Torkamani, 2019). Complex systems like ML and DL models make predictions without explaining their logic or decision-making process. These models may damage professional and patient confidence and cause ethical and legal issues. We must make ML and DL models more understandable and interpretable (Papadimitroulas et al., 2021; Rasheed et al., 2022). Although these technologies have revolutionized oncology by providing more accurate diagnoses, treatment predictions, and survival estimates, they are still in development and require validation through more research and clinical trials (Xiao et al., 2022).

In this study, we conducted an in-depth review of the current implementations of AI subfields, namely ML and DL, in the context of GI cancer research. Specifically, we focused on the use of these techniques for early detection, diagnosis, prediction of therapy response, and survival analysis by using different input data in various studies, regarding the present obstacles and constraints encountered in the field of GI cancers.

## 2 The difference between AI in research and clinical applications

AI has touched many sectors, including medicine. AI has many uses in research and therapy, but the path from AI research to clinical AI should be clarified (Yin et al., 2021; Shaheen, 2021).

Researchers mostly use AI to analyze massive datasets, spot patterns, and generate hypotheses. Academics use AI to analyze complex datasets. AI systems analyze vast datasets to discover problem-solving solutions quicker than conventional approaches. The AI model may identify patterns and links that could assist human researchers in identifying any open gaps. AI is growing in the study because it automates monotonous jobs and yields rapid results (Shaheen, 2021; Shao et al., 2022).

In therapeutic applications, life and death are at stake, with crucial moments. Healthcare AI algorithms must meet various requirements and verify their dependability, safety, and effectiveness. AI in health care is like a close buddy who helps detect issues. AI is used in clinical settings to identify illnesses by analyzing medical pictures, test findings, and patient history. AI may create a tailored treatment plan for a patient with particular traits and a medical history. Patient surveillance: AI may monitor patients and notify clinicians of noteworthy changes (Yin et al., 2021; Haleem et al., 2019; Iqbal et al., 2021).

Translating research into clinical practice requires several steps:

1. Validation and testing: AI systems must undergo rigorous validation and testing processes to function in healthcare contexts.
2. US regulators, like the FDA, approve AI systems first. This process explains AI system safety and effectiveness.
3. Clinical studies: Establish AI’s operational efficiency in a clinical environment and its benefits and hazards.
4. Clinical workflow integration: AI technology should help healthcare staff rather than replace them.
5. Training and education: Medical practitioners should be taught about AI technology and its constraints.
6. Continuous monitoring and enhancement: To ensure optimal operation and address emerging concerns before and after system upgrades, AI systems must undergo regular reviews.

In conclusion, AI may revolutionize healthcare, but we should not forget that research and clinical situations are different. A series of thorough validations can ensure the safe adoption of AI technology in labs and hospitals (Yin et al., 2021; Shaheen, 2021; Shao et al., 2022; Haleem et al., 2019; Iqbal et al., 2021; Rakha et al., 2021).

## 3 AI’s role in GI cancers

**Figure 1:**
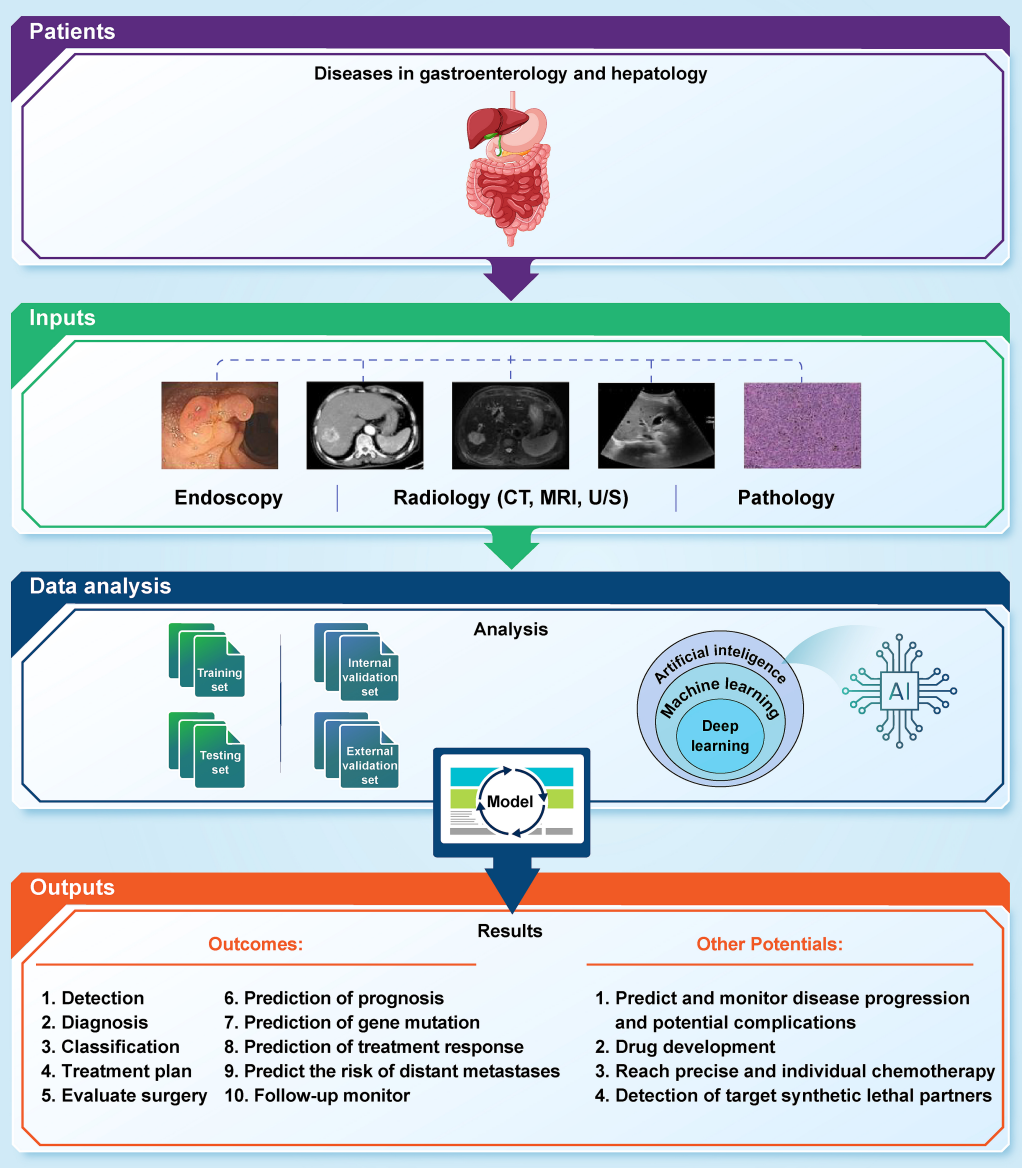
Application of artificial intelligence in various GI cancers.

We provide an in-depth examination of how artificial AI is changing the field of GI cancer. Gastrointestinal malignancies pose distinct difficulties, requiring accurate diagnostic instruments and tailored therapeutic approaches. Utilizing AI, namely ML and DL algorithms, has shown significant promise in transforming early detection, diagnosis, treatment planning, and prognosis of GI cancers (Figure 1). Several recent studies highlight the ability of AI to uncover complex patterns in large volumes of data, resulting in valuable insights that improve the accuracy of early diagnoses, the effectiveness of treatments, and the accuracy of estimating the survival rate of GI cancer patients (Mukherjee et al., 2024; Pooja, 2024; Ahn and Shah, 2024; Wong et al., 2022; Tabari et al., 2022). We highlight the profound influence of AI in the field of GI cancer, signaling a fundamental shift towards more accurate and focused cancer treatment. The result highlights the need for continuous research endeavors and cooperative endeavors among AI researchers, healthcare practitioners, and policymakers. By promoting multidisciplinary cooperation, we can effectively navigate the ever-changing field of GI cancer treatment. This will allow us to fully use the potential of AI to enhance patient outcomes and contribute to a more efficient and individualized approach to managing GI cancers (Table 1).

**Table 1:** Previous studies assessing AI’s role in gastrointestinal cancers.

Authors	Topic	Cancer types	Training data	Techniques	Reported outcomes
Hashimoto et al. (2020)	Artificial intelligence using convolutional neural networks for real-time detection of early esophageal neoplasia in Barrett’s esophagus (with video)	Esophageal cancer	Endoscopic images	CNN	Per-image accuracy: 95.4%; Per-image Sn/Sp: 96.4%/94.2%; 98.6%/88.8% (WLI); 92.4%/99.2% (NBI)
Struyvenberg et al. (2020)	Improved Barrett’s neoplasia detection using computer-assisted multiframe analysis of volumetric laser endomicroscopy.	Esophageal cancer	Endoscopic images	PCA-CAD	AUC of Multi-frame: 0.91; AUC of Single-frame: 0.83
van der Putten et al. (2020)	Deep principal dimension encoding for the classification of early neoplasia in Barrett’s Esophagus with volumetric laser endomicroscopy	Esophageal cancer	Endoscopic images	Multi-step PDE-CNN on an A-line basis	AUC: 0.93; F1 score: 87.4%
Ohmori et al. (2020)	Endoscopic detection and differentiation of esophageal lesions using a deep neural network. Gastrointestinal endoscopy	Esophageal cancer	Endoscopic images	CNN	Ac/Sn/Sp: 77%/100%/63% (Non-ME+NBI/BLI); 81%/90%76% (Non-ME +WLI); 77%/98%/56% (ME)
Guo et al. (2020)	Real-time automated diagnosis of precancerous lesions and early esophageal squamous cell carcinoma using a deep learning model (with videos)	Esophageal cancer	Endoscopic images	CNN-SegNet	Per-image Sn/Sp: 98.04%/95.03%; Per-frame Sn/Sp: 91.5%/99.9%
Kloeckner et al. (2020)	Multi-categorical classification using deep learning applied to the diagnosis of gastric cancer	Gastric cancer	Gastric cancer images	CNN	ROC curves above 0.9
Shibata et al. (2020)	Automated detection and segmentation of early gastric cancer from endoscopic images using mask R-CNN	Gastric cancer	Endoscopic images	RNN	Dice index = 71% Sn = 96%
Leon et al. (2019)	Automated detection and segmentation of early gastric cancer from endoscopic images using mask R-CNN	Gastric cancer	Histopathological Samples	Deep-CNN	Detection accuracy = 89.72%
Sakai et al. (2018)	Automatic detection of early gastric cancer in endoscopic images using a transferring convolutional neural network	Gastric cancer	Endoscopic images	CNN	Detection accuracy = 82.8%
Thapa et al. (2019)	Using machine learning to predict progression in the gastric precancerous process in a population from a developing country who underwent a gastroscopy for dyspeptic symptoms	Gastric cancer	Gastroscopy samples	Random Forest	Sn = 86% Sp = 79%
Kang et al. (2020)	Automatic three-dimensional cephalometric annotation system using three-dimensional convolutional neural networks: a developmental trial	Hepatocellular carcinoma	CT images	Neural Network Fuzzy Neural Network	Ac = 79.19%
Das et al. (2019)	Deep learning based liver cancer detection using watershed transform and Gaussian mixture model techniques	Hepatocellular carcinoma	CT images	DNN	Ac = 99.38%
Kumar et al. (2022)	A systematic review of artificial intelligence techniques in cancer prediction and diagnosis. Archives of Computational Methods in Engineering	Hepatocellular carcinoma	CT images	SVM	Ac = 98%
Gruber et al. (2019)	A joint deep learning approach for automated liver and tumor segmentation. 2019 13th International conference on Sampling Theory and Applications (SampTA)	Hepatocellular carcinoma	CT Liver images	DNN	Ac = 99.9%
Chlebus et al. (2018)	Automatic liver tumor segmentation in CT with fully convolutional neural networks and object-based postprocessing	Hepatocellular carcinoma	CT images	Deep-CNN	Detection rate = 77%
Hoerter et al. (2020)	Current treatment options in gastroenterology	Colorectal cancer	ImageNet database	CNN	Per-polyp sensitivity = 71%
Shin et al. (2018)	Automatic colon polyp detection using region based deep CNN and post learning approaches. IEEE Access	Colorectal cancer	Polyp images and videos	Deep-CNN	Detection processing time = 0.39 s
Godkhindi and Gowda (2017)	Automated detection of polyps in CT colonography images using deep learning algorithms in colon cancer diagnosis	Colorectal cancer	CT images	CNN	Polyp detection accuracy = 88%
Zhang et al. (2016)	Automatic detection and classification of colorectal polyps by transferring low-level CNN features from nonmedical domain	Colorectal cancer	Endoscopic images	CNN	Ac = 85.9% Precision = 87.3% Recall = 87.6%
Yamada et al. (2019)	Development of a real-time endoscopic image diagnosis support system using deep learning technology in colonoscopy	Colorectal cancer	Polyp images and videos	Deep learning	Sp = 97.3%
Sinkala et al. (2020)	Machine Learning and Network Analyses Reveal Disease Subtypes of Pancreatic Cancer and their Molecular Characteristics	Pancreatic cancer		ANN	AI in differentiation of two PDAC subtypes: overall classification Ac 100% for the mRNA-based model, 99% for the DNA methylation model; model provides predictions of clinical response to chemotherapy
Muhammad et al. (2019)	Pancreatic Cancer Prediction Through an Artificial Neural Network.	Pancreatic cancer		ANN	AI Sn and Sp in testing cohort: 80.7, 80.7%; AUROC curve 0.85
Corral et al. (2019)	Deep Learning to Classify Intraductal Papillary Mucinous Neoplasms Using Magnetic Resonance Imaging.	Pancreatic cancer		ANN	AI in detect dysplasia Sn and Sp: 92, 52%. Identification of high-grade dysplasia/cancer: Sn and Sp 75 and 78% AI AUROC curves 0.78 (*p* = 0.90) vs. AUROC base on AGA criteria 0.76, AUROC based on Fukuoka criteria 0.77
Săftoiu et al. (2015)	Differential diagnosis of PDAC and CP using CHEUS and TIC analysis	Pancreatic cancer	Endoscopic ultrasound images	ANN	AI Sn Sp PPV and NPV using TIC analysis on CH-EUS: 94.64, 94.44, 97.24, 89.47%
Zhang et al. (2010)	Differential diagnosis of PDAC from normal tissue (based on 29 pattern features)	Pancreatic cancer	Endoscopic ultrasound images	SVM	AI Ac, Sn, Sp, PPV and NPV for the diagnosis of pancreatic cancer: 97.98, 94.32, 99.45, 98.65, 97.77%

EUS, endoscopic ultrasound; AI, artificial intelligence; PDAC, pancreatic adenocarcinoma; CP, chronic pancreatitis; F1-score, FNA fine needle aspiration; ANN, artificial neural network; CNN, convolutional neural network; Ac, accuracy; Sp, specificity; Sn, sensitivity; PPV, positive predictive value; NPV, negative predictive value; TIC, time-intensity curve; AUROC, area under the receiver operating characteristic; AUC, area under the curve; AUC, Area under ROC curve; RNN, Regional-based NN; SVM, Support vector machine; VLE, Volumetric laser endomicroscopy; WLI, White light imaging.

### 3.1 Esophageal cancer

Esophageal cancer (EC) is known as the second most lethal GI cancer and the sixth most frequent cause of cancer-related mortality worldwide (Allemani et al., 2018). Two primary histological subtypes of EC with distinctive clinicopathological features include esophageal adenocarcinoma (EAC) and esophageal squamous cell carcinoma (ESCC) (Li et al., 2022a). The diagnosis of EC entails several techniques, including histopathology, CT scan, MRI scan, positron emission tomography (PET) scan, endoscopy, and biopsy (Lowe et al., 2005; Van Rossum et al., 2016). In spite of advanced technologies in diagnostic and therapeutic strategies, the survival rate of EC patients is low (Huang and Yu, 2018; Choi et al., 2022; Tsuji et al., 2023). Chemotherapy, chemoradiotherapy, immunotherapy, and targeted therapy served as possible treatment options for patients declared inoperable (Valkema et al., 2023; Li J. J. et al., 2023; He et al., 2022). The treatment options for this cancer include Carboplatin/Paclitaxel, Cisplatin/5-Fluorouracil (5-FU), Epirubicin/Cisplatin/5-FU (ECF), and Docetaxel/ Cisplatin/5-FU (DCF) (Abraham et al., 2021).

In the context of early detection of EC, ML and DL techniques have been established to be supportive in analyzing various kinds of data, including genomic, transcriptomic, methylation, and histopathologic data. These approaches are aimed at discovering biomarkers and risk factors associated with EC (Li J. et al., 2023). In a recent study conducted by Allegra et al. (2022), the authors elucidated the use of DL techniques in the analysis of omics data and the calculation of genomic changes from histopathology pictures. ML and DL have also shown encouraging potential in the field of medical image processing, namely in computer vision. These advancements have proven valuable in aiding healthcare professionals in making diagnostic decisions that are both more precise and faster (Islam et al., 2022). The mean accuracy of these techniques in the analysis of endoscopic and CT images of the esophagus exceeded 89%, suggesting a significant influence on the timely identification of esophageal cancer (Hosseini et al., 2023a). In a previous study conducted by Zhang P. et al. (2022). DL has been created for the purpose of analyzing barium esophagrams, which are cost-effective diagnostic tests used for the detection of EC. DL model demonstrated a detection accuracy of 90.3%, a sensitivity of 92.5%, and a specificity of 88.7% for the detection of esophageal cancer. Convolutional neural networks (CNNs), in contrast to other ML techniques, have shown superior accuracy and sensitivity in the early diagnosis of EC, according to recent thorough research (Hosseini et al., 2023a). Recently, some studies have looked at how well ML algorithms can be used to find EC early on using non-invasive methods like measuring blood, saliva, and breath (Liu Q. et al., 2023; Valkema et al., 2021).

Traditional techniques like biopsy, histopathology, and endoscopy are invasive, subjective, and take a long time (Xie et al., 2021). As a result, more precise, effective, and non-invasive techniques for EC diagnosis are required. ML and DL techniques have the potential to aid in the diagnosis of EC by effectively classifying the tumor’s type, stage, and grade using endoscopic images, CT images, and histopathological images. These strategies have the potential to enhance the precision and effectiveness of EC diagnosis, while also minimizing inter-observer variability and reducing human mistakes (Karahan Şen et al., 2021; Tomita et al., 2019; Hosseini et al., 2023b). Previous studies surveyed the recent applications of ML and DL models for the diagnosis of EC based on genomic, transcriptomic, and proteomic data (Li M.-X. et al., 2021; Chen Z. et al., 2021). Li M.-X. et al. (2021) work introduced stratifin as an ideal prognostic biomarker for ESCC via the use of ML methods. Stratifin, encoded by SFN, was discovered as the most effective prognostic biomarker in three separate groups of patients with ESCC. It can distinguish between ESCC patients with varying clinical outcomes. Their investigation revealed that the shared frequencies across various feature selection methods indicate the level of significance, with the highest-ranked one being the key molecule with clinical relevance. A recent investigation not only showed the metabolites that can be used for diagnosis and prognosis but also identified potential targets for treating ESCC. Their approach used a combination of metabolomics data and ML algorithms to develop a new strategy for diagnostic tool creation. The results demonstrated that ML models based on metabolites had accurate and consistent predictive performance, which encourages the future use of ML in analyzing metabolomics data. Additionally, the study found that disturbed amino acid metabolism, including the accumulation of essential amino acids and increased expression of amino acid transporters, is a significant characteristic of ESCC. Their study also identified SLC1A5, a specific amino acid transporter, as a potential target for anti-ESCC treatment (Chen Z. et al., 2021). Kumagai et al. (2019) used DL to examine endocytoscopic pictures of the esophagus to ascertain whether AI may assist endoscopists in substituting biopsy-based histology. A DL model using CNN architecture, namely GoogLeNet, was developed. The model was trained using a dataset consisting of 4,715 photos of the esophagus, with 1,141 images classified as malignant and 3,574 images classified as non-malignant. To assess the diagnostic precision of the AI, a separate group of 1,520 endocytoscopic pictures, obtained from 55 consecutive patients (27 with ESCCs and 28 with benign esophageal lesions), were analyzed. Based on the examination of the receiver-operating characteristic curve (ROC curve), the area under the receiver operating characteristics curve (AUC) for the total images, higher magnification pictures, and lower magnification pictures were 0.85, 0.90, and 0.72, respectively. The AI accurately identified 25 out of the 27 instances of ESCC, resulting in an overall sensitivity rate of 92.6%. Out of the 28 non-cancerous lesions, 25 were correctly identified as non-malignant. The specificity of this diagnosis was 89.3% and the total accuracy was 90.9%. The AI misinterpreted two examples of malignant lesions as non-malignant, while the endoscopist accurately classified them as malignant. Out of the three instances, where non-cancerous abnormalities were incorrectly identified as carcinogenic by the AI system, two cases were diagnosed as radiation-related esophagitis and one case was diagnosed as gastroesophageal reflux disease. The researchers reported that AI has the potential to assist endoscopists in detecting ESCC using endocytoscopic pictures, without relying on biopsy-based histological reference.

As mentioned, ML and DL techniques have the potential to contribute to the prediction of therapy response in EC. This may be achieved by integrating diverse data types, including radiomic, imaging, genomic, and clinical data. By doing so, a more thorough and holistic understanding of EC can be obtained (Xie et al., 2021; Wang J. et al., 2022; Lukomski et al., 2023). Sheng et al. (2023) developed a novel prediction model named HybridNet, which used a DL network and integrated clinical variables with dose information to achieve precise prediction of radiation pneumonitis (RP) after irradiation. The HybridNet model demonstrated superior performance compared to ML-based and dosiomics-based models, as well as the ResNet model while using simple dosage matrices as input. It successfully enhanced the ability to forecast the occurrence of RP, indicating its potential as an appreciated tool for making therapeutic decisions. Recent studies have also elucidated the potential use of ML and DL techniques in predicting the efficacy of neo-adjuvant chemoradiotherapy (nCRT) based on CT and endoscopic images of patients with EC (Hu et al., 2021; Kawahara et al., 2022). As an example, Hu et al. (2021) accompanied a retrospective study in which they included patients with ESCC from April 2007 to December 2018. The patients were selected from two different institutions. The researchers obtained DL characteristics from six pre-trained CNNs by analyzing pretreatment CT scans in the training cohort, which consisted of 161 cases. The classifier used was the support vector machine (SVM). Validation was conducted using a separate testing group consisting of 70 individuals. The researchers evaluated the performance by using AUC and identified an ideal model. This model was then compared to a radiomics model that was generated using the training cohort. A clinical model, comprised only of clinical characteristics, was also constructed for baseline comparison. They further performed a radiogenomics investigation using gene expression patterns to uncover the underlying biology linked to radiological prediction. The ResNet50-based model produced an AUC and accuracy of 0.805 (95% CI, 0.696–0.913) and 77.1% (65.6–86.3%) respectively in the testing cohort. In comparison, the radiomics model achieved an AUC and accuracy of 0.725 (0.605–0.846) and 67.1% (54.9–77.9%) respectively. The radiological models exhibited superior prediction accuracy compared to the clinical model. The researchers determined that the unique and noninvasive deep learning method might provide a precise and efficient prediction of treatment response to nCRT in ESCC. They suggest that the innovative and noninvasive DL technique might accurately predict ESCC treatment response to nCRT and aid clinical decision making.

Recently, scientists revealed how ML and DL may predict the survival of EC patients. They discovered that ML and DL may give prognostic information and risk classification for EC patients, as well as identify variables that impact the survival outcome (Wang J. et al., 2022; Lukomski et al., 2023). Nunez et al. (2023) conducted a prognostic research aims to construct and assess neural natural language processing models for the purpose of predicting the survival outcomes of patients diagnosed with general cancer. This prediction was based on the analysis of their first oncology consultation document. The models use several language representations, including CNNs, bag-of-words, long short-term memory networks, and bidirectional encoder representations from transformers. The research demonstrates that the models are capable of achieving a notable level of accuracy, surpassing conventional ML techniques. Recently, researchers also presented a multimodal DL design that uses CT scans and clinical data to predict the survival of EC patients. They showed that their approach outperformed the usual techniques that employ just one type of data. A DL survival network demonstrated more promising findings in predicting EC-specific survival than the tumor-node-metastasis (TNM) staging system (Huang et al., 2022). A novel staging method, known as DeepSurv, was designed by Zhang et al. (2023) using a DL technique and the SEER database. The purpose of this system was to improve the precision of overall survival prediction specifically for patients diagnosed with ESCC. A noninvasive prediction model was also developed for EC by Wang J. et al. (2022) that uses a combination of noninvasive techniques, including DL-based radiomics (DLR) features, handcrafted features, and clinical characteristics which aims to predict survival rates within a three-year timeframe from the time of diagnosis. The DLR nomogram outperformed the standard radiomics model in terms of Harrel’s concordance index and the AUC. The calibration curves demonstrated the excellent predictive capability of the nomogram. The Kaplan–Meier survival (KMS) curves predicted by the nomogram showed a significant difference compared to the nonsurvival groups, as shown by the log-rank test (*p*-value <0.05). The suggested approach provided physicians with a foundation to enhance treatment options and tailor diagnoses to individual patients.

The use of ML and DL techniques shows potential for enhancing the practical application of evidence-based medicine of EC. These approaches provide the potential to improve clinical decision-making, aid in the early detection, diagnostic process, advice for therapy selection and facilitate the prediction of patient outcomes (Huang S. et al., 2020; Xie et al., 2021). Hence, it is essential to encourage more cooperation and communication among researchers, medical professionals, patients, and policymakers in order to promote the integration of ML and DL methodologies into clinical practice, while simultaneously ensuring their safety and effectiveness.

### 3.2 Gastric cancer

Gastric cancer (GC) is an oncological condition characterized by malignant growth originating from the inner mucosal layer of the stomach. The majority of instances of stomach malignancies are classified as gastric carcinomas, which may be further categorized into several subtypes, such as gastric adenocarcinomas (WCRF International, 2022). It ranks as the fifth most prevalent kind of cancer on a global scale (WCRF International, 2022). According to global estimates, the total number of mortality attributed to GC in the year 2020 was around 768,793 individuals (American Society of Clinical Oncology (ASCO), 2023). On a global scale, it is recognized as the fourth most prominent contributor to mortality in cancer cases. The prevalence of stomach cancer is at its highest in the eastern Asian region, particularly in China, Cabo Verde, Bhutan, and Tajikistan (International Agency for Research on Canver (IARC), 2022). The standard treatment approach for GC often includes a combination of many therapeutic modalities, such as surgical intervention, chemotherapy, and radiation therapy (Li G. Z. et al., 2022). The choice of treatment is dependent upon the patient’s general health, severity, and stage of the condition. The main therapy for resectable GC is radical surgery, or gastrectomy. Before or after surgery, some patients may get radiation treatment and/or chemotherapy. The standard treatment for metastatic GC is systemic chemotherapy. The intricate biology of GC has led to the ineffectiveness of specific treatments, save for trastuzumab, which targets HER2, and ramucirumab, which targets VEGFR2. Immunotherapy and biomarker-directed treatments have also shown significant advancements (Wang Y. et al., 2022; Xu et al., 2023). Recently, AI, including ML and DL techniques, has shown encouraging outcomes in the timely detection, diagnosis, prognostication of treatment efficacy, and overall survival rates pertaining to GC (Jamil et al., 2022; Zhao et al., 2022).

AI has shown its potential in the screening of GC by effectively identifying precancerous conditions and aiding in the early detection of cancer via the use of endoscopic examination and confirmation through pathological analysis (Cao et al., 2022). ML and DL have the potential to assist in the diagnosis of gastric cancer by providing assistance for tumor-node-metastasis (TNM) staging and subtype classification (Cao et al., 2022). Recently, researchers designed a cost-effective, non-intrusive, efficient, and accurate diagnostic model using six ML algorithms. The purpose was to categorize patients into high or low-risk categories for the development of GC based on the analysis of individual lifestyle factors. The research identified eleven significant characteristics that impact the incidence of GC, including *Helicobacter pylori* infection, excessive salt consumption, chronic atrophic gastritis, and other factors (Afrash et al., 2023). Hirasawa et al. (2018) devised a CNN capable of autonomously identifying GC in endoscopic images. The CNN-based detecting system was built using the Single Shot MultiBox Detector architecture. It was trained on a dataset of 13,584 endoscopic images specifically focused on GC. The generated CNN was tested using an independent set of 2,296 stomach photos obtained from 69 consecutive individuals with 77 GC lesions to assess its diagnosis accuracy. The CNN took 47 s to examine a total of 2,296 test pictures. The CNN accurately identified 71 out of 77 GC lesions, yielding an overall sensitivity rate of 92.2%. Additionally, 161 non-cancerous lesions were mistakenly classified as GC, resulting in a positive predictive value of 30.6%. A total of 98.6% of the 71 lesions with a diameter of 6 mm or larger, as well as all invasive malignancies, were accurately identified. All the lesions that were not detected were shallowly depressed and were intramucosal tumors of the differentiated type. These cancers were challenging to differentiate from gastritis, even for experienced endoscopists. Approximately 50% of the false-positive lesions were identified as gastritis, characterized by alterations in color tone or an irregular mucosal surface. The developed CNN system for GC detection can efficiently analyze a large number of stored endoscopic images within a short timeframe while maintaining clinically significant diagnostic accuracy. Implementing this approach in daily clinical practice can effectively alleviate the workload of endoscopists. Another investigation was also carried out to do a thorough evaluation, including scholarly articles that used AI-based learning algorithms for the purpose of detecting. A total of 110 studies have identified that using both conventional ML and DL-based classification methodologies is important for GC detection (Bhardwaj et al., 2022). Overall, these approaches possess the capability to initiate the preliminary screening of GC and identify patients at a heightened risk, hence necessitating more invasive examinations (Afrash et al., 2023). These novel techniques have the potential to significantly reduce the incidence of instances requiring endoscopic monitoring. Nevertheless, it is crucial to acknowledge that while these methodologies exhibit potential, more research is necessary to confirm the effectiveness of the models in a broader and multicenter cohort (Afrash et al., 2023).

ML and DL approaches are becoming more prevalent in the field of GC diagnosis, with promising outcomes in terms of accuracy and efficacy. Especially, the field of medical image analysis has seen a significant surge of interest in DL techniques, mostly owing to their ability to provide results that are on par with, and in many instances, even superior to those achieved by professionals (Gonçalves et al., 2020). This has contributed to the growing prominence of DL in the medical domain. Recent papers offer the considerable potential and limitations seen in DL research projects pertaining to GC, ulcers, gastritis, and non-malignant diseases (Gonçalves et al., 2020). Recent retrospective multicenter study that conducted by Huang et al. (2021), introduced an accurate diagnosis and prognosis prediction model for GC using DL on digital pathological images, known as GastroMIL. This model demonstrated a discriminatory capacity with an accuracy of 0.920 in the external validation set, greater to that of the junior pathologist and comparable to that of expert pathologists. Zhu et al. (2022) also introduced a novel framework that was developed by integrating statistical approaches and DL techniques to investigate the associations between GC and tongue traits. This framework also plays a significant role in facilitating the efficient early detection of individuals diagnosed with GC. A recent retrospective research aimed to create an inexpensive, quick, non-invasive, and high-precision GC diagnosis model utilizing personal behavioral lifestyles and non-invasive features by integrating 3,630 individuals. The created models (extreme gradient boosting, random forest, decision tree, and logistic regression) were tested by cross-validation and the generalization ability in their test set. They discovered that the model constructed utilizing fingerprints based on the extreme gradient boosting (XGBoost) technique gave superior outcomes compared with the other models. The total accuracy of the test set was 85.7%, AUC was 89.6%, sensitivity 78.7%, specificity 76.9%, and positive predictive values 73.8%, demonstrating that the proposed model has high medical value and strong practical prospects (Jiang et al., 2022).

Additionally, numerous studies have used ML and DL techniques to predict the treatment response among individuals diagnosed with GC. The main focus of an investigation by Wang et al. (2018) was to examine the use of DL techniques in the analysis of several omics data types, including genomic, methylation, and transcriptomic data. Additionally, the research explores the application of DL in histopathology-based genomic interpretation. The article additionally discusses the integration of various data types in the development of decision-support tools for cancer diagnosis, prognosis, and treatment management. The latest comprehensive study presents encouraging findings in the use of deep learning techniques for predicting the efficacy of drug treatments in cancer patients such as GC. This study explores various data formats, neural network topologies, learning procedures, and assessment systems. Additionally, this study conducts a comparative analysis of deep learning models and traditional machine learning models, revealing that deep learning models exhibit superior performance and provide more favorable outcomes (Partin et al., 2023). Chen Y. et al. (2021) created and verified a predictive model for determining the primary pathological response to neoadjuvant chemotherapy (NAC) in patients with advanced GC (AGC) using a ML algorithm. ML techniques were used to pick radiomic characteristics from venous-phase CT images in order to construct a radscore. In conjunction with other clinical factors identified via univariate analysis, the radscores were included in binary logistic regression analysis in order to develop a comprehensive prediction model. The data acquired for the validation cohort were used to assess the prediction precision of the model. The researchers constructed and tested a prediction model that included adenocarcinoma differentiation and radscores. The model facilitates the stratification of individuals based on their susceptibility to NAC and has the potential to function as a tool for personalized therapy decision-making in patients with AGC. Jiang et al. (2023) provide a novel methodology for noninvasively predicting the tumor microenvironment (TME) status using radiological imaging. Their strategy involves the integration of radiomics and DL analyses by using cohorts consisting of 2,686 individuals diagnosed with GC. Their study demonstrates that the radiological model effectively predicts the state of TME and serves as an independent prognostic factor, surpassing the predictive capabilities of clinicopathologic factors. The model also predicts the potential advantages of adjuvant treatment for individuals diagnosed with localized GC. The prediction accuracy of clinical response in patients undergoing checkpoint blockade immunotherapy is enhanced by the integration of the model with validated biomarkers. The methodology used in this study allows for the noninvasive evaluation of TME, hence facilitating the possibility of longitudinal observation and assessment of the effectiveness of GC treatment.

Numerous studies have been established to investigate the use of ML and DL techniques in the prediction of survival outcomes among individuals diagnosed with GC. Recently, Zeng et al. (2023) constructed models using DL survival neural networks in order to predict the survival outcomes of patients diagnosed with gastric adenocarcinoma. Additionally, the study sought to evaluate the predictive capabilities of these models by comparing them to other established survival models often used in clinical settings. The study contained a cohort of 14,177 patients diagnosed with gastric adenocarcinoma, obtained from the Surveillance, Epidemiology, and End Results (SEER) database. These patients were randomly assigned to either the training or testing group, with a ratio of 7:3. The prediction models were constructed using two selected methods, namely random survival forest (RSF) and a DL-based survival prediction algorithm called DeepSurv. The patients with GC were divided randomly into two groups: a training group consisting of 9,923 patients and a testing group consisting of 4,254 patients. DeepSurv exhibited higher performance compared to the traditional CoxPH model and the RSF with a 3-year survival prediction model. DeepSurv achieved a c-index of 0.772 and an IBS of 0.1421, while the CoxPH model achieved a c-index of 0.755 and an IBS of 0.1506, and the RSF model achieved a c-index of 0.766 and an IBS of 0.1502. The DeepSurv model demonstrated exceptional accuracy and precise survival estimates for predicting survival rates at 1, 3, 5, and 10 years, with an AUC ranging from 0.825 to 0.871. The DeepSurv model exhibits superiority over the CoxPH and RSF models, demonstrating excellent discriminative performance and calibration. Another research was conducted by Jung et al. (2023) with the objective of establishing a cost-effective, not disruptive, efficient, and accurate diagnostic model. This model used six ML algorithms to classify patients into high or low-risk categories for the development of GC. The classification was based on the analysis of various lifestyle characteristics of individual patients. A different study used clinical characteristics, radiomics features, and DL features discovered by CNNs in order to forecast the overall and progression-free survival rates of individuals diagnosed with GC using multi-modal data from 1,061 patients, 743 for model learning and 318 independent patients for evaluation. Clinical factors and CT imaging characteristics extracted by radiomics and DL were used to develop a Cox proportional-hazard model for overall and progression-free survival prediction. Clinical, radiomics, and DL features were further examined for prediction. The concordance index (c-index) was the model performance indicator, while pre-and post-operative hazard ratios (HRs) assessed multi-modal feature prediction effects. Cox’s multi-modal hazard predicts survival for 318 independent testing group patients. For overall and progression-free survival prediction, the greatest c-index was 0.783 (95% CI, 0.782–0.783) and 0.770 (95%, 0.769–0.771). The post-operative variables are significantly (*p* < 0.001) more predictive than the pre-operative variables. Significant survival predictors include tumor stage, lymph node stage, carcinoembryonic antigen (CEA), chemotherapy treatment, radiomics signature, and DL signature (HR = 1.336/1.768, *p* < 0.005). The study found that CT radiomics and DL imaging characteristics are strong pre-operative predictors, supplementing pathological staging indicators. Lower CEA levels and chemotherapy improve survival. They suggested that these findings improve GC prognosis and therapy planning (Hao et al., 2022). Various studies have shown the considerable potential of ML and DL techniques in enhancing the accuracy of early detection, diagnosis, treatment response, and survival prediction for patients diagnosed with GC (Jamil et al., 2022; Jin et al., 2020; Jiang et al., 2021). This advancement has significant promise in facilitating informed clinical decision-making processes.

### 3.3 Hepatocellular carcinoma

Hepatocellular carcinoma (HCC) is the prevailing liver cancer (Mitra, 2011). In terms of mortality ranking, HCC has the position of being the fourth most prominent cause of cancer-related mortality on a global scale (Dasgupta et al., 2020; Chen et al., 2022). HCC frequently manifest in individuals with chronic liver diseases such as chronic infection with specific hepatitis viruses, DNA mutation in liver cells, and risk factors including cirrhosis, certain inherited liver diseases, diabetes, hepatic steatosis, and excessive alcohol consumption (Mitra, 2011; Dasgupta et al., 2020). The conventional therapeutic modalities for HCC include hepatectomy, liver transplantation, ablation techniques, radiation therapy, immunotherapy, and targeted pharmacotherapy (Mitra, 2011). The majority of HCC patients will undergo various types of chemotherapy with the aim of extending their lifespan. Sorafenib is the initial molecular inhibitor authorized by the FDA for the management of advanced HCC. Before sorafenib became available, doxorubicin was commonly administered as a monotherapy for advanced HCC. However, it has demonstrated ineffectiveness, with a response rate of approximately 15–20%. Additional chemotherapeutic drugs, including epirubicin, cisplatin, 5-fluorouracil, etoposide, and their combinations, exhibit even less effectiveness (Cao et al., 2012). The survival rates for HCC have seen improvement throughout the years, perhaps because of advancements in early detection methods and therapy strategies (Ding and Wen, 2021). As an example, individuals diagnosed with early-stage liver malignancies who undergo liver transplantation have a 5-year survival rate ranging from 60 to 70% (Ghishan and Kiela, 2011).

The use of ML and DL techniques has shown encouraging outcomes in the timely identification of hepatocellular carcinoma (HCC). A recent research which conducted by Zhang et al. (2020), used a computational methodology using ML techniques to analyze a collection of microarray data derived from 1,091 HCC samples and 242 samples obtained from individuals without HCC. This research used the within-sample relative expression orderings (REOs) technique to derive quantitative descriptors from datasets including gene expression profiles. By using the maximum redundancy minimum relevance (mRMR) technique with incremental feature selection, the researchers successfully eliminated irrelevant information. This process led to the identification of an “11-gene-pair” that exhibited exceptional performance. This developed computer model demonstrates the ability to differentiate between HCC and adjacent non-cancerous tissues, even when dealing with limited biopsy specimens or specimens that have been improperly collected. This model has promise for assisting in the early detection of HCC at an individual level (Zhang et al., 2020). A further investigation introduced a novel technique known as No End-repair Enzymatic Methyl-seq (NEEM-seq) (Deng et al., 2023), which effectively detects methylation with high accuracy while minimizing DNA damage. The researchers made further advancements in their study by introducing a read-level neural detection model known as DeepTrace. This model exhibits improved capabilities in accurately identifying sequencing reads originating from HCC by using a pre-trained and fine-tuned neural network. This model demonstrated a high level of accuracy (96.2%), sensitivity (93.6%), and specificity (98.5%) in the validation cohort, which included 62 patients with HCC, 48 patients with liver disease, and 20 healthy persons. These results were obtained using the whole-genome NEEM-seq data of cell-free DNA. During the first phase of HCC, the DeepTrace method exhibited a sensitivity of 89.6 and 89.5%, exceeding the sensitivity of Alpha Fetoprotein (AFP) which showed much worse performance (Deng et al., 2023). These results underscore the promise of AI techniques in enhancing the timely detection of HCC.

ML and DL also have shown considerable promise in the diagnosis of HCC. In a recent study, Shen et al. (2023) conducted a comprehensive analysis of previous and ongoing studies on the use of conventional models and techniques in AI applications related to serology, imaging, histology, proteomics, and genetic diagnosis of HCC. Menegotto et al. (2021) developed an algorithm using multimodal DL methods to help in the computerized diagnosis of HCC. This system integrates preprocessed CT images with structured data extracted from patients’ Electronic Health Records (EHRs). Recent findings indicate that DL recurrent neural network (RNN) models exhibited superior performance compared to traditional logistic regression (LR) models, implying the potential use of RNN models in accurately identifying individuals with hepatitis C virus (HCV)-related cirrhosis who are at a heightened risk of developing HCC. The researchers utilized raw longitudinal data directly retrieved from EHRs, with conventional regression models in predicting the likelihood of getting HCC. This study examined the prognosis of 48,151 patients diagnosed with cirrhosis caused by HCV in the national Veterans Health Administration. The patients were followed up for at least 3 years after the diagnosis of cirrhosis. Patients were selected based on their positive HCV RNA test results. They were then monitored from the time of cirrhosis diagnosis, to observe any new cases of HCC. Three models were created and tested to predict HCC over a 3-year period. These models include: (1) LR with cross-sectional inputs (cross-sectional LR); (2) LR with longitudinal inputs (longitudinal LR); and (3) RNN with longitudinal inputs. The findings of this study indicate that DL-RNN models were more effective than traditional LR models in identifying patients with HCV-related cirrhosis who are at a high risk of developing HCC. This suggests that RNN models could be utilized for risk-based HCC outreach and surveillance methods (Ioannou et al., 2020). The process of diagnosing primary liver cancers, specifically HCC and cholangiocarcinoma (CC), is difficult and requires a significant amount of time and effort, even for professionals. The presence of secondary liver cancers adds more complexity to the diagnosis. AI provides effective answers to these diagnostic problems by enabling the histological categorization of cancers utilizing digital whole slide images (WSIs). The objective of a recent study was to create a sophisticated DL algorithm capable of accurately differentiating between HCC, CC, and metastatic colorectal cancer (mCRC) based on histological images. Additionally, the study aims to explore the potential clinical consequences of this model. The WSIs obtained from HCC, CC, and mCRC were utilized for training the classifiers. The AUCs for HCC, CC, and mCRC were 0.989, 0.988, and 0.991, respectively, in the classification of normal and malignant cases. The HCC/other cancer type classifier was trained using appropriate tumor samples to accurately differentiate between HCC and CC and mCRC. The combined AUC value of the classifier was 0.998, indicating high effectiveness. Afterwards, the CC/mCRC classifier accurately distinguished between CC and mCRC with a combined AUC of 0.995. Nevertheless, evaluation on an independent dataset demonstrated that the HCC/other cancer type classifier exhibited suboptimal performance, with an AUC of 0.745. By merging the initial training datasets with additional external datasets and undergoing retraining, the classification was significantly enhanced, resulting in all reaching perfect AUCs of 1.000 (Jang et al., 2023). A comprehensive study was also conducted to analyze several ML and DL techniques for the detection of chronic liver disease and HCC. This research offers a full overview of the ML pipeline, including pre-processing, feature extraction, and learning algorithms (Singh et al., 2023). These studies emphasize the potential of ML and DL techniques in enhancing the diagnosis and treatment of HCC.

Numerous researches have been conducted to investigate the function of ML and DL in the prediction of treatment response in HCC. Zou et al. (2021) conducted a comprehensive study that demonstrated the valuable contribution of ML algorithms in predicting the therapeutic success of patients with HCC after different treatment modalities. They emphasized the efficacy of ML algorithms in forecasting treatment results and discussed the difficulties associated with ML algorithm selection during the construction of a model. Retrospective cohort research had a sample size of 605 individuals diagnosed with intermediate-stage HCC who underwent transcatheter arterial chemoembolization (TACE) as their primary treatment modality. The digital subtraction angiography (DSA)-Net framework comprises two models: Model 1, which called U-net model used for the automated segmentation of tumors, and Model 2, which named ResNet model applied for predicting the treatment response to the first TACE. The two models underwent training on a dataset consisting of 360 patients. Subsequently, an internal validation was performed on a separate group of 124 patients, while an external validation was conducted on an additional 121 cases. The performance of Models 1 and 2 was assessed using the Dice coefficient and receiver operating characteristic curves, respectively (Zhang L. et al., 2022). A proof-of-concept investigation assessed the application of ML to forecast the likelihood of recurrence based on laboratory, clinical, and MRI data collected before therapy in patients with early-stage HCC who were initially suitable for liver transplant. This retrospective study comprised a cohort of 120 patients diagnosed with early-stage HCC who initially qualified for liver transplantation and received therapy by transplantation, resection, or thermal ablation. Patients underwent pre-treatment MRI and post-treatment imaging surveillance. The study used pretreatment clinical variables (including laboratory data) and extracted imaging features to construct three ML models (clinical model, imaging model, combined model) to predict recurrence within 6 years following therapy. During the follow-up period, a tumor reoccurred in 44 out of 120 patients (recurrence rate of 36.7%). Briefly, the results indicated that ML algorithms could forecast the likelihood of recurrence in patients with early-stage HCC who are primarily suitable for liver transplantation, even before treatment is assigned. Incorporating MRI data into the model input significantly enhanced the predicted accuracy compared to relying just on clinical criteria. The combined model did not exceed the performance of the imaging model (Iseke et al., 2023). This kind of results illustrates the potential of AI approaches in offering important tools for personalized therapy approaches.

Recently, scientists revealed how ML and DL may predict the survival of HCC patients. As previously noted, in a recent research, researchers conducted a study whereby they devised a DL model to predict the likelihood of HCC recurrence subsequent to resection or liver transplantation. A recent study offered a prognostic classifier, based on DL models, on histological slides obtained from patients with HCC. This classifier aids in enhancing the prognostic prediction of HCC patients and identifies individuals who have derived benefits from more intensive therapy strategies. The model was constructed using a sample size of 1,118 patients derived from four distinct cohorts that were independent of each other. The model underwent extensive evaluation across diverse patient populations undergoing various treatment modalities, constantly demonstrating exceptional performance in assessing classical clinical, biochemical, and pathological characteristics. The suggested technique, which utilizes CNNs, has the potential to enhance the assessment of patient prognosis and provide valuable guidance to physicians when considering the implementation of adjuvant treatment for their patients (Liu Z. et al., 2022). An additional research endeavor was undertaken to construct a prediction model for microvascular invasion (MVI) utilizing DL techniques. The objective was to provide visual explanations for the model’s predictions, thereby facilitating its implementation in clinical settings. The performance of the attention-guided multi-phase fusion network in predicting preoperative MVI was outstanding. The most prominent sites that contribute to the prediction of MVI are the tumor margins in the four stages and the peritumoral areas in the arterial and hepatobiliary phases. Among the four stages, the Human Brain Project (HBP) made the most significant contribution to the prediction of MVI. The visualization of attention weights in the training network enhances the comprehensibility of the underlying causal connection between deep features and MVI. This, in turn, augments the interpretability of DL models in clinical settings, potentially streamlining the adoption of DL techniques in clinical practice. The precise preoperative estimation of microvascular invasion (MVI) may aid in the identification of patients with HCC who are susceptible to MVI. This can assist healthcare professionals in making informed decisions on the most appropriate treatment strategies, ultimately leading to improved patient survival outcomes (You et al., 2023).

### 3.4 Colorectal cancer

Colorectal cancer (CRC), often referred to as bowel or colon cancer, frequently originates as a polyp inside the colon or rectum. Over the course of time, some polyps have the potential to undergo malignant transformation, leading to the development of cancer (Centers for Disease Control and Prevention, 2023a). CRC ranks as the fourth most prevalent cause of mortality caused by cancer. The overall five-year survival rate for colon cancer is reported to be 64.6%. However, it is important to note that survival rates might vary depending on the stage of the disease. Specifically, the five-year survival rate for stage 1 colon cancer can reach as high as 92%, while for colorectal cancer at any other stage, the survival rate can be as low as 34.9% (Centers for Disease Control and Prevention, 2023b; Mangone et al., 2022). Hence, timely detection of CRC via comprehensive screenings, through conventional methods and novel pertinent molecular biomarkers, is crucial for the successful implementation of treatment strategies and the enhancement of overall patient prognosis (Kanth and Inadomi, 2021; Eshkiki et al., 2022). The standard treatment modality for CRC often entails surgical intervention aimed at the excision of the malignant tumor. Additional therapeutic interventions, such as radiation therapy and chemotherapy, may be advised based on the cancer’s location and stage of the malignancy (Mayo Clinic, 2023a). CRC treatment options include targeted medicines, chemotherapy, and immunotherapy. The selection of treatment modalities is mostly determined by the cancer’s stage (American Cancer Society, 2023). The standard therapy for advanced CRC involves the administration of a combination of 5-FU and leucovorin together with either oxaliplatin or irinotecan. The introduction of monoclonal antibodies like Bevacizumab and Cetuximab has significantly advanced the medical therapy of CRC. Although response rates have improved with the use of other modulation techniques, such as combined treatment using monoclonal antibodies and conventional chemotherapy, around 50% of patients with metastatic CRC still show resistance to chemotherapies based on 5-FU (Van der Jeught et al., 2018).

In the past few years, AI methodologies have shown significant potential in the timely detection of CRC. The objective of a recent study was to use DL techniques in order to develop models for the detection, localization, and classification of colorectal lesions using white light endoscopic images. The researchers gathered and organized the endoscopic images captured in white light from a cohort of individuals who had colonoscopies. The use of CNNs model is employed for the purpose of identifying the presence of lesions in an image, specifically those pertaining to CRC, colorectal adenoma (CRA), and colorectal polyps. The model’s performance is assessed using measures such as accuracy, sensitivity, and specificity rates. Subsequently, the instance segmentation model is used to accurately identify and categorize the lesions present in the images including such abnormalities. The model’s performance is then assessed using metrics such as mean average precision (mAP), AP50, and AP75. These metrics serve as evaluative measures for the effectiveness of the instance segmentation model. The researchers devised and conducted a comparative analysis of five DL models in order to identify lesions in white light endoscopic pictures. According to the findings, the ResNet50 network design demonstrated the most favorable performance. Additionally, the Mask R-CNNs model illustrated the capability to accurately identify and categorize lesions within images that include such anomalies (Gao et al., 2020). Recently, a different study discovered that an ANN model had superior performance as the most effective algorithm in predicting CRC and non-CRC characteristics. The researchers reached the conclusion that a fusion of unsupervised and supervised ML methodologies may be used to investigate the fundamental dietary characteristics for the purpose of predicting CRC. In order to enhance feasibility and practicality, it was determined that the ANN algorithm exhibited optimum performance, achieving a misclassification rate of 1% for CRC cases and a misclassification rate of 3% for non-CRC cases. This finding suggests that using the ANN algorithm may significantly improve the efficacy of cancer screening techniques. Additionally, they noted that the use of dietary information as a non-invasive approach for screening purposes has the potential to be implemented on a wide scale among large populations. The findings also indicate that the use of optimum algorithms in conjunction with a high level of adherence to cancer screening protocols will have a substantial impact on enhancing the effectiveness of CRC prevention (Abdul Rahman et al., 2023). Some recent comprehensive studies also discussed how CRC, is classified according to DL and ML techniques. These findings underscore the promise of ML and DL techniques in enhancing the timely detection and diagnosis of CRC (Yin et al., 2023; Tharwat et al., 2022).

When using metagenomic data for the diagnosis of CRC, it is often observed that DL methods generally exhibit worse performance in comparison to standard ML approaches for the prediction and diagnosis of this cancer especially when utilizing metagenomic data to identify this cancer. However, Thanh-Hai et al. introduced a methodology that employs manifold learning techniques such as t-distributed stochastic neighbor embedding (t-SNE) and spectral embedding to convert numerical data into visual representations. This approach also incorporates DL algorithms to enhance the accuracy and effectiveness of predicting CRC diseases. The study also presented significant opportunities for enhancing the quality and efficiency of visualizations in predictive tasks involving dense data. The findings of the analysis conducted on samples obtained from five distinct areas, namely America, China, Austria, Germany, and France, demonstrate the potential of integrating visualization techniques with DL algorithms to improve the accuracy and effectiveness of diagnosing CRC (Thanh-Hai and Thai-Nghe, 2020). In a separate investigation, scholars developed a categorization framework with the aim of distinguishing between five distinct categories of lung and colon tissues. These categories included two benign kinds and three malignant types, and the differentiation was achieved via the analysis of histological images. The findings obtained demonstrated that the suggested framework has the capability to accurately detect cancer tissues with a maximum accuracy of 96.33%. It is believed that the use of this model will assist healthcare practitioners in the creation of an automated and dependable system with the ability to accurately detect different forms of lung and colon cancers (Masud et al., 2021). Sakr et al. (2022) proposed a lightweight CNN-based DL technique for effective colon cancer diagnosis. The effectiveness of the suggested method was examined using histopathology images and compared to current techniques in the area. Results indicated the suggested technique for colon cancer diagnosis was more susceptible and efficient than the previous deep models. Their model achieved the highest accuracy, precision, recall, and F1-score (99.50, 99, 100, and 99.49%). In circumstances when pathologists require to be insured to check colon images, their approach may aid in accurate diagnosis. Zhou et al. (2020) devised a DL framework named CRCNet to facilitate the optical detection of CRC. The model was trained using a dataset consisting of 464,105 colonoscopic imaging data obtained from 12,179 patients. To evaluate its efficacy, the performance of CRCNet was assessed on a separate cohort of 2,263 patients, drawn from three distinct datasets. The findings of the study indicate that CRCNet has a notable capability to effectively distinguish CRC from benign conditions, including adenomas and polyps, with a commendable level of accuracy. The CRCNet model demonstrated consistent and strong performance in accurately identifying patients with CRC across three separate test sets. The performance of the subject in question exhibited similarity to that of a cohort consisting of five proficient endoscopists. However, a variety of comprehensive studies acknowledged that while AI technologies show promise, there is still much to learn about their use in clinical settings, therefore more research is required to fully actualize these technologies’ potential (Tharwat et al., 2022; Tamang and Kim, 2021).

Recently, scientists revealed how ML and DL may predict the treatment response of CRC patients. A recent comprehensive investigation conducted an examination of research findings pertaining to the four most frequent and often occurring malignancies globally, namely lung, breast, prostate, and CRC. Subsequently, a novel approach for the detection of CRC using SBERT and SimCSE sentence representations was introduced. The only input for this technique consisted of raw DNA sequences obtained from matched tumor/normal pairs of CRC. The acquired representations were then used as input for ML classifiers in order to perform classification. Based on the evaluation of the ML classifiers, it was determined that XGBoost had the highest performance among all classifiers. Additionally, the use of SimCSE representations resulted in very minimal enhancements in the classification efficacy of the ML models. In their study, Kong et al. emphasized the significance of using predictive biomarkers to classify CRC patients, since this approach is crucial for enhancing the efficacy of anti-cancer medication treatments and ultimately improving therapeutic results. The researchers used a ML framework in their study, which aimed to uncover reliable pharmacological biomarkers. This framework utilized network-based analyses and relied on pharmacogenomic data obtained from three-dimensional organoid culture models. The scientists have successfully found biomarkers using their methodology, which have shown a high level of accuracy in predicting the treatment responses of a cohort consisting of 114 patients with colorectal cancer who were treated with 5-fluorouracil, as well as 77 patients with bladder cancer who were treated with cisplatin. The study introduced a novel methodology for predicting the response of cancer patients to drugs. This approach utilizes pharmacogenomic data obtained from organoid models and combines the use of gene modules with network-based methods (Mokoatle et al., 2023). The availability of analytical and bioinformatics approaches has facilitated the use of high-dimensional data, allowing for the emergence of high-throughput phenotyping. Additionally, the existence of dynamic models has enabled the connection of phenomena across various levels, ranging from genes to cells, cells to organs, and ultimately, throughout the entire organism. In this context, D’Orazio et al. (2022) introduced a novel, entirely automated system capable of extracting meaningful data on the dynamic nature of cellular morphological phenotypes. This system successfully establishes a strong link between gene expression and cellular phenotype. The researchers used phenomics, including image analysis, DL, and ML techniques, to develop a comprehensive methodology known as ML-Phenomics (MLP). The efficacy of the proposed MLP platform has been evaluated in terms of its capability to discern phenotypic traits of cells associated with the downregulation of the LOX-1 receptor (gene expression pathway) or the administration of drugs in a dose-dependent manner (drug effects pathway) in CRC Cells. The findings indicate that the suggested platform exhibited superior performance compared to other benchmark approaches, regardless of the specific neural network used. Patients diagnosed with locally advanced rectal cancer (LARC) stand to benefit significantly from the attainment of a pathological complete response (pCR) subsequent to nCRT, as it offers them the most favorable prognosis. The effectiveness of an ANN model in predicting pathological complete response (pCR) in patients with locally advanced rectal cancer (LARC) was assessed by Huang C.-M. et al. (2020). The study included a comparison of the predictive performance of several models, including ANN, k-nearest neighbor (KNN), SVM, naïve Bayes classifier (NBC), and multiple logistic regressions (MLR). The performance of the forecasting models was compared using data obtained from a sample of 270 individuals with LARC. The findings of their study demonstrated that the post-chemoradiotherapy carcinoembryonic antigen (CEA) level is the primary predictor of pCR, with subsequent predictors including the duration between chemoradiotherapy and surgery, the kind of chemotherapy regimen, the clinical nodal stage, and the clinical tumor stage. The ANN model demonstrated superior accuracy in predicting pCR compared to other traditional prediction models. Zhu et al. (2021) developed a DL model using magnetic resonance imaging (MRI) to predict the response of tumors in patients diagnosed with CRC liver metastasis (CRLM). This particular model demonstrated a high level of efficacy in predicting the pathological tumor response to preoperative chemotherapy and long-term survival following hepatectomy. Furthermore, the researchers assert that this model exhibited greater accuracy compared to the RECIST criteria, which currently serve as the predominant criteria for evaluating the clinical response of solid tumors to chemotherapy. The identification of microsatellite instability (MSI) in CRC is of utmost importance in clinical decision-making, as it enables the identification of individuals who exhibit varying therapy responses and prognoses. In a recent study, Yamashita et al. (2021) conducted an investigation into the feasibility of using a DL-based system to automate the prediction of MSI directly from whole-slide images (WSIs) stained with Hematoxylin and Eosin (H&E). The DL model, known as MSINet, was constructed utilizing a dataset consisting of 100 WSIs stained with H&E. These WSIs were obtained from patients who underwent primary CRC resection, with a total of 343 patients in the pool. The selection of WSIs was performed in a class-balanced manner, with 50 WSIs exhibiting microsatellite stability (MSS) and the other 50 WSIs exhibiting MSI. The WSIs were scanned at a magnification of 40×. The DL model demonstrated superior performance compared to experienced GI pathologists in predicting MSI on H&E-stained WSIs. They reported such a model has the potential to serve as an automated screening tool for triaging individuals for confirmatory testing. This might lead to a reduction in the number of patients who need to be tested, resulting in significant savings in terms of labor and costs associated with testing. Numerous recent comprehensive studies have also shown the potential and positive outcomes of ML and DL in enhancing the prediction of treatment response in CRC (Qiu et al., 2022; Partin et al., 2023; Tran et al., 2021).

In recent years, there has been widespread use of ML and DL models for the purpose of predicting the survival outcomes of patients diagnosed with CRC. DL algorithms have shown effective use in the analysis of H&E stained WSIs for the purpose of predicting patient outcomes, including overall survival, progression-free survival, time to metastasis, and tumor recurrence. Li et al. (2022b) proposed a unique method called DeepDisMISL, which is based on distribution and designed for multiple-instance survival learning. The purpose of their study was to investigate the hypothesis that the inclusion of comprehensive patch information in WSIs might enhance the accuracy of predicting CRC survival. Rather than only using patches with the highest and lowest scores, the researchers opted to use patches that were evaluated according to their placement within the percentile distributions. The researchers provided evidence that this particular methodology has the potential to significantly enhance the accuracy of prognostic predictions for patients with CRC, hence improving their survival outcomes. Incorporating several examples of neighboring areas around each chosen distribution point, such as percentiles, has the potential to enhance the accuracy of predictions. DeepDisMISL exhibited superior predictive performance and more precise risk stratification for overall survival in comparison to the six state-of-the-art baseline methods, as shown in both the MCO CRC and TCGA COAD-READ datasets. The DeepDisMISL model demonstrated a high level of interpretability, allowing for the identification of connections and interdependencies between morphological features and the risk of cancer prognosis in patients. According to the study conducted by Buk Cardoso et al. (2023) AI models have shown their effectiveness in accurately predicting the survival rates of patients diagnosed with CRC. This prediction was based on data obtained from hospital-based cancer registries in low and middle-income countries, with a particular focus on the XGBoost algorithm. This research conducted five distinct classes, taking into account the survival of patients. The ML models yielded predictions and identified the key aspects of each method utilized in the conducted research. The predictive models yielded an accuracy rate of around 77%, with an AUC value approaching 0.86. Notably, the clinical staging variable consistently emerged as the most influential factor across all models. The study conducted by Kather et al. (2019) aimed at evaluating the capability of deep CNNs in identifying prognostic indicators directly from HE-stained tissue slides, which are commonly accessible images of CRC patients. The findings of this retrospective research demonstrated that CNN has the capability to evaluate the human tumor microenvironment and make prognostic predictions based only on histological pictures. The research emphasizes the potential of ML and DL techniques in enhancing the accuracy of survival rate predictions for individuals diagnosed with CRC. This advancement has the potential to significantly enhance treatment options and eventually improve patient outcomes.

### 3.5 Pancreatic cancer

Pancreatic cancer (PC) is regarded as one of the most lethal forms of cancer, with a very low percentage of survival. Ranked as the fifth leading cause of cancer-related mortality, this particular malignancy has a five-year survival rate that falls below 7% (Pancreatic Cancer UK, 2023). The decision about the course of therapy for pancreatic cancer is contingent upon the specific stage of the cancer’s progression and the general health condition of the patient. The conventional treatment options for this condition are surgical intervention, chemotherapy administration, and radiation therapy (Mayo Clinic, 2023b; Shokati Eshkiki et al., 2022). Surgical procedures may include the partial or complete excision of the pancreas, perhaps necessitating the removal of adjacent organ structures. Additional therapeutic interventions for symptom management may include the use of endoscopy. Chemotherapy relies on various medications, such as 5-Fluorouracil, Capecitabine, Irinotecan, and Oxaliplatin. This treatment modality may be used to manage symptoms in cases where surgical intervention is not feasible, to prevent cancer recurrence following surgical procedures, to reduce tumor size prior to surgery, or to address early-stage cancer. Radiotherapy may be used for the management of incipient neoplastic conditions or in cases where surgical intervention is recommended due to the patient’s severe debilitation or the cancer cannot be removed by surgery (Mayo Clinic, 2023b; NHS, 2023).

Within the field of medicine, AI methodologies have made significant contributions to advancements in the study and prediction of a diverse range of cancers. The use of these models in the early detection of PC is seeing a growing trend. These methodologies have the potential to identify people with an increased susceptibility to PC, hence potentially leading to a better rate of survival among patients afflicted with this condition (Gupta et al., 2022). Scientists at Harvard Medical School and the University of Copenhagen devised an AI tool that accurately detected individuals with the greatest susceptibility to PC, even up to three years before diagnosis, by only analyzing the patient’s medical data. The researchers used AI techniques to analyze clinical data from a cohort of 6 million patients, including 24,000 individuals diagnosed with PC. The researchers used ML models to train on the sequence of disease codes seen in clinical histories. They then examined the capacity of these models to predict the presence of cancer within certain time windows (CancerRiskNet). This research was conducted with the intention of explicitly including the chronological order of disease occurrences and evaluating the capacity to predict the risk of cancer over progressively longer time intervals between the endpoint of the disease trajectory used for risk prediction and the onset of cancer. The findings suggest that using the chronological order of disease histories as input to the model, as opposed to only considering disease incidence at any given time, enhances the predictive capability of AI techniques in estimating the onset of PC, particularly for those in the highest-risk group (Placido et al., 2023). Malhotra et al. (2021) conducted a research by using EHR from the UK to study a cohort of over 1,000 individuals ranging in age from 15 to 99 years who had a diagnosis of PC. The researchers devised an algorithm that acquired the ability to differentiate between individuals who subsequently got PC and those who did not. By using this methodology, it was shown that 41% of patients who were below the age of 60 were classified as being at a high risk level, as much as 20 months previous to their diagnosis. The researchers have successfully shown their findings have the potential to enhance early detection by using a multi-stage screening framework that incorporates newly discovered biomarkers, specifically targeting those deemed to be at a heightened risk based on this methodology. In a separate investigation done by Dinesh et al. (2023), the objective was to anticipate the occurrence of PC at an early stage via the evaluation of medical imaging data, specifically CT scans. The researchers used CNN and YOLO model-based CNN (YCNN) models to detect crucial characteristics and cancerous growths inside the pancreas. This research introduces an automated YCNN model as a tool to assist pathologists in the classification of PC grades using pathological pictures. Additionally, the YOLO model was included in the system to provide predictions based on the available data. The images were resized and segmented into patches of 224 × 224 pixels before being simultaneously inputted into the YCNN. Subsequently, CNN was used to classify the patches according to their corresponding grades. Then, the patches were reassembled to provide a unified picture, which is then sent to the pathologist as the ultimate outcome. The reported f1-scores on the datasets are 0.99 and 1.00, indicating very favorable results. They reported the study results have the potential to aid pathologists in establishing a uniform diagnosis for the grading of PC. Liu Y. et al. (2023) introduced an innovative ANN model that has considerable potential in the realm of clinical practice, namely in the timely identification of PC. The ANN model has shown remarkable proficiency in reliably discriminating PC samples from normal samples and proficiently forecasting the attributes of previously unseen samples. By using a comprehensive bioinformatics analysis, the researchers conducted a thorough investigation into the expression patterns of pancreatic cancer-specific miRNAs (PCSMs). Furthermore, they successfully clarified the relationships between these PCSMs and other clinical features. The study revealed notable associations between PCSMs and age, thereby emphasizing their potential significance in the fields of pharmaceutical testing, individualized therapeutic strategies, and immunotherapy for PC. Park et al. (2022) have created a DL system to incorporate longitudinal clinical data from EHR in order to estimate the risk for PC. The researchers highlighted that their approach to masking yielded significant improvements in distant time periods leading up to the diagnosis. Additionally, their “grouped” neural network (GrpNN), displayed improved generalizability by mitigating the issue of overfitting compared to the feedforward baseline model. The findings exhibited consistency across self-reported racial categories. In the field of healthcare, ML and DL algorithms have shown their effectiveness as a feasible means of classifying or detecting the risk of PC, thereby leading to enhanced rates of survival (Gupta et al., 2022).

The latest developments underscore the potential of AI techniques to enhance the diagnosis and treatment of PC (Katta et al., 2023). ML algorithms may be used to identify people with an elevated risk of getting PC at an earlier stage, leading to a better rate of survival among patients (Malhotra et al., 2021). The use of ML and DL in the domain of pancreatic disease imaging is seeing tremendous growth. These techniques may be used for the identification of pancreatic ductal adenocarcinoma (PDAC) and other types of PC, as well as for the characterization of pancreatic lesions (Barat et al., 2021). Karar et al. (2023) devised a novel and effective model, one dimensional-convolutional neural networks-long short-term memory (1D CNNs-LSTM), specifically tailored for the multi-classification of patients with PC. This model utilizes urine biomarkers that have been carefully selected for their distinctive characteristics. The categorization findings classify the status of the pancreas into three categories: healthy pancreas, benign instances, and PDAC cases. The generated model attained the utmost values of assessment criteria, notably a 97% accuracy, surpassing existing ML and CNN-based models. Implemented CNN models, both with and without the LSTM layer, successfully accomplished precise identification of the tested PDAC samples. This study combined created 1D CNN-LSTM with a real urine microfluidics device to perform real-time clinical trials on urine samples from patients with PC. They mentioned the Internet of Medical Things (IoMT) technology may be used in this area of research to provide a mobile-based automated diagnosis of patient samples via medical cloud services. Tong et al. (2022) did research with the goal of creating a DL radiomics (DLR) model using contrast-enhanced ultrasound (CEUS) images. The objective was to help radiologists accurately distinguish between PDAC and chronic pancreatitis (CP). A cohort of 558 patients with pancreatic lesions was included in the study and divided into four groups: the training cohort, the internal validation cohort, and two external validation cohorts. The DLR model exhibited greater sensitivity and specificity compared to the diagnoses made by the five radiologists in the three validation cohorts. The use of the DLR model resulted in enhanced diagnostic sensitivity across all radiologists in the three validation cohorts, with a little or negligible reduction in specificity. Al-Fatlawi et al. (2021) have shown that the combination of deep sequencing and ML may greatly enhance the accuracy of PC detection using blood samples. The researchers demonstrated that by combining the well-established biomarker CA19-9 with novel, reliable RNA-based variations, they were able to accurately distinguish between PC and pancreatitis, achieving an impressive AUC of 96%. The genes B4GALT5 and GSDMD, which include three very important variations, are strongly associated with the advancement of cancer and the increase in CA19-9 levels. In addition, they discovered six variations that were shown to have a statistically significant association, with one of them being established as a breast cancer risk factor in many publications. Their findings demonstrated that the combination of deep sequencing and ML has the potential to provide timely and precise diagnosis, as well as individualized treatment alternatives. This combined technique also established a set of 16 important variations that may accurately predict survival in resectable PDAC. These variants were able to distinguish between resected PC and chronic pancreatitis with an AUC of 96%.

Several researches have investigated the function of AI in predicting the treatment response in cases of PC. Recent studies have shown that ML-based methods have potential applications in pancreatic surgery. These techniques can be used to accurately diagnose pancreatic conditions before surgery, evaluate prognosis and predict complications after surgery (Skawran et al., 2021; Palumbo et al., 2021; Li X. et al., 2021; Lee et al., 2021). Multiple comprehensive research studies have shown promising outcomes in treatment selection, with an emphasis on combining omics and histopathology data. These studies also emphasize the integration of various kinds of data and the use of DL approaches to predict cancer response to medication therapies (Partin et al., 2023; Tran et al., 2021; Preuss et al., 2022). Radiomics (Parr et al., 2020; Cozzi et al., 2019) and DL (Watson et al., 2021) are two quantitative imaging technologies that use data science and current medical imaging. Recently, these methods have shown growing potential in enhancing the precision management of PC via personalized therapy and optimization. Parr et al. (2020) utilized radiomic models using pretreatment CT scans to predict the overall survival and local recurrence of PC after stereotactic body radiation therapy. The radiomic model and the model that combines radiomic and clinical parameters performed better than the pure clinical model in making these predictions. The average concordance index for survival was 0.66 and 0.68 for the radiomic models, compared to 0.54 for the pure clinical model. Similarly, the average AUC for recurrence was 0.78 and 0.77 for the radiomic models, compared to 0.66 for the pure clinical model. Cozzi et al. (2019) successfully used a hybrid clinical-radiomics model to accurately distinguish between patients at high and low risk in terms of overall survival after treatment with stereotactic body radiation therapy. In order to assess the predictive capability of a radiomics signature in determining the clinical outcome of patients with PC who underwent stereotactic body radiation therapy (SBRT), they conducted a retrospective analysis on a group of 100 patients. Radiomics texture features were extracted from CT images of the clinical target volume. The patient cohort was randomly divided into two groups for training (60 patients) and validation (40 patients) purposes. Cox regression models were constructed to forecast overall survival and local control. A CT-based radiomic signature was identified, which exhibited a correlation with both overall survival and local control after SBRT. This signature enabled the identification of low and high-risk patient groups. The model achieved an impressive AUC value of 0.81. Neoadjuvant chemotherapy (NAC) has the potential to enhance the survival rate of patients diagnosed with pancreatic adenocarcinoma. Nevertheless, accurately assessing the effectiveness of this therapy is challenging. Watson et al. (2021) used DL-CNN to predict the pathological tumor response to NAC in PC. Patients undergoing NAC before pancreatoduodenectomy were found in cases of PC. The institution is known as the College of American Pathologists. The classification of tumor regression grades 0–2 corresponds to a pathologic response (PR), whereas grade 3 indicates no response (NR). Preoperative CT scans were used to generate axial images to construct a 5-layer convolutional neural network and LeNet-DL model, to predict PRs. The hybrid model resulted in a 10% reduction in carbohydrate antigen 19–9 (CA19-9). Their model achieved an AUC of 0.738 in predicting the response to chemotherapy and an accuracy of 78.3% in predicting the response to resectability. They noted the model’s performance is enhanced by including reductions in serum CA19-9 levels as a factor and this neural network can accurately forecast the pathological tumor response to NAC in patients diagnosed with pancreatic adenocarcinoma.

Recent evidence emphasizes the capacity of ML and DL to enhance the accuracy of predicting survival rates in PC. This advancement has the potential to result in improved treatment choices and more effective planning of care requirements (Barat et al., 2021; Preuss et al., 2022). A researcher conducted an assessment of the efficacy of ML in predicting survival outcomes, comparing it to the TNM approach and published nomograms. PC patients were discovered using the Surveillance, Epidemiology, and End Results database (SEER). The clinical data of the patients was retrieved and then separated into two sets: a training set, including 80% of the records, and a validation set, comprising the remaining 20%. ML techniques were evaluated for predicting the likelihood of survival at 6, 12, and 24 months, in addition to the TNM staging system and two nomograms. The model achieved an AUC of 86.6% at 6 months, 83.4% at 12 months, and 82.2% at 24 months. The TNM staging method obtained an AUC of 66.6% at 6 months, 65.5% at 12 months, and 57% at 24 months, in comparison. The nomograms demonstrated an AUC of 71.1 and 55.2% in accurately predicting 1-year survival. ML outperformed both the TNM staging method and prognostic nomograms (Osman, 2019). Lee et al. (2022) created a DL model called the PC-survival model. This model applies a general adenocarcinoma feature extractor (GAFE) to estimate the probability of mortality based on the histomorphological aspects of adenocarcinoma lesions. Their model for predicting survival in PC was trained using WSIs stained with H&E. In addition, to assess the ability to apply knowledge to new situations, they use the PC survival model to make predictions about the mortality risk associated with rectum adenocarcinoma and breast adenocarcinoma. All of the data studied in this research was obtained exclusively from The Cancer Genome Atlas (TCGA). A self-supervised contrastive learning approach was used to train GAFE using a set of 328, randomly selected, adenocarcinoma H&E stained WSIs. To assess the survival model for PC, they used 5-fold cross validation. The C-index mean for the TCGA-PAAD test datasets was 0.7258; the maximum and minimum values were 0.7784 and 0.6598, respectively. They assessed mortality risk using the model with median performance (C-index: 0.7216) and classified patients into 50% high-risk and 50% low-risk groups. From this, the log-rank test’s *p*-value was 0.01986. They combined the top five models to examine the generalization’s performance. They used both TCGA-READ and TCGA-BRCA to evaluate their combined model. Whereas the C-index for TCGA-BRCA was 0.5711, it was 0.6941 for TCGA-Read. It was discovered that the histomorphological characteristics of adenocarcinoma were somewhat related to the prognosis of survival in PC.

## 4 AI obstacles in GI cancers

The clinical use of AI (ML and DL) in GI cancers has several concerns and obstacles. These include issues related to the quality and quantity of data since ML and DL models need substantial volumes of high-quality data for effective training. Another significant difficulty pertains to their interpretability. ML models, particularly those using DL techniques, are often regarded as ambiguous entities because of the inherent challenge of comprehending their predictive mechanisms. The absence of transparency might impede the acceptability of these entities by physicians (Montavon et al., 2018). These models often exhibit a characteristic known as “black box” behavior, which poses challenges for physicians in comprehending the underlying mechanisms behind these models’ predictive capabilities. These models should be capable of offering straightforward and intelligible reasons for their predictions (Papadimitroulas et al., 2021; Rasheed et al., 2022). Furthermore, variations in the interpretation of medical images by different physicians may have an impact on the accuracy of AI techniques (Niu et al., 2020). The efficacy of ML models may be impeded by the restricted expertise and absence of objective standards in the interpretation of medical images (Cao et al., 2022). To address the issue of limited data availability and enhance the precision of ML models, it is essential to establish a framework for standardized data collection and uniform processing protocols (Wang S. et al., 2023). Additionally, the absence of established legal and ethical frameworks, together with the absence of user-friendly and interactive interfaces, should also be taken into consideration. Numerous researchers encounter challenges related to class imbalance and biases, emphasizing the need to validate detection algorithms across organizations in longitudinal investigations (Hosseini et al., 2023a). There are several obstacles and possible solutions to consider including data curation and privacy. The bottleneck is in the curating of extensive, top-notch, annotated datasets that are accessible to the public. The curation of imaging data is a costly and laborious procedure that requires specialized knowledge in imaging anatomy and pathology. Furthermore, there are logistical and ethical considerations around the ownership of data and the safeguarding of patient privacy that might impede the creation and dissemination of public imaging datasets (Larson et al., 2020; Prevedello et al., 2019). The consideration of generalizability is also crucial since it presents a significant challenge, given that models trained on data from a specific population or healthcare system may exhibit worse performance when applied to other populations or systems. ML algorithms that have been trained on datasets consisting of older individuals and limited sample sizes may exhibit worse performance when applied to larger populations in real-world scenarios (Larson et al., 2020; Prevedello et al., 2019). Initial endeavors in using a CNN for the identification of PC showed significant potential; however, more external validation using datasets from multiple institutions is required (Larson et al., 2020; Prevedello et al., 2019; Park et al., 2020). Overall, integrating ML and DL methods into the current healthcare workflow may provide some obstacles (Abdul Rahman et al., 2023; Tharwat et al., 2022; Alboaneen et al., 2023; Bychkov et al., 2018).

## 5 AI future advancement/perspective in GI cancers

Notwithstanding the obstacles, the outlook for AI’s future application in the context of GI cancers seems to be encouraging. ML and DL models have the potential to enhance the precision and effectiveness of GI cancer screening and detection. The use of these models has the potential to facilitate the creation of individualized treatment strategies that are tailored to the specific attributes of each patient. These models have the potential to enhance the accuracy of predicting patient outcomes and survival rates. ML-based prediction models, such as the dietary-based prediction model, provide a solid foundation for the development of efficient clinical decision support systems. These systems aid medical professionals in non-invasive screening linked to dietary factors, hence facilitating their use in extensive research projects. The use of these approaches offers substantial assistance in the detection of the first phases of cancer, hence facilitating prompt intervention and resulting in a reduced death rate in contrast to the rate seen after the manifestation of symptoms (Abdul Rahman et al., 2023; Tharwat et al., 2022; Alboaneen et al., 2023). Initial endeavors in using a CNN for the identification of PC showed significant potential; however, more external validation using datasets from multiple institutions is required (Li X. et al., 2021; Lee et al., 2021; Preuss et al., 2022). For optimal results, all patients included in the external data should come from the same institution. This ensures that any technological variations between the two institutions do not impact the categorization process (Chu et al., 2019a; Chu et al., 2019b). There is a need for AI models that can be easily understood and interpreted. It is crucial to get confidence from physicians and comprehend the fundamental patterns that the models are acquiring (Tran et al., 2021). Further investigation is required to address these obstacles and fully realize the capabilities of AI in the domain of GI cancers. This includes enhancing the precision of detection and classification models, devising models capable of managing substantial amounts of data, and investigating novel treatment approaches derived from the prognostications of those models (Ahn et al., 2021).

## 6 Conclusion

This study highlights the capacity of AI techniques, namely ML and DL, in early detection, diagnosing, predicting treatment response, and analyzing survival rates of GI tumors. The growing need and increasing complexity of diagnosing GI cancer have led to the integration of digital pathology into the diagnostic process. Digital pathology enables the acquisition, management, and interpretation of pathology data in a digital setting, hence creating possibilities for computational analysis with AI. Several ML techniques demonstrate encouraging sensitivity and specificity in the diagnosis of GC. The SVM technique, in particular, shows exceptional resilience and capacity for generalization. DL, using intricate algorithms to mimic the human brain network, is a potent tool in digital pathology. We have provided a concise overview of the performance attributes and constraints of fully supervised and weakly supervised methods concerning the tasks of classifying, segmenting, detecting, and predicting the progression of GI cancers. We provided clinical perspectives on how AI might aid in the timely identification of lesions, categorization of tumors, and extensive cancer screening. To fully harness the benefits of AI, it is crucial to address the significant obstacles associated with algorithm creation. These problems include dealing with the diverse nature of histological image data, managing the heterogeneity in interpretations among physicians, and ensuring model transparency and interpretability in clinical situations. This predicament remains unresolved. Ensuring acceptable standards of AI requires rigorous external validation and quality controls, supported by an enormous dataset, from a clinical viewpoint. This necessitates ongoing research on model design, focusing on patch/pixel-level annotation, explainability, and generalizability of AI algorithms, while also considering the heterogeneity of multiethnic populations. Our study has rigorously compared the current AI model for GI cancer and has identified the challenges in developing AI for clinical analysis. We have provided a comprehensive summary of the existing AI algorithms developed for GI cancers and have highlighted clinical insights for the future development of GI cancer AI algorithms. As AI algorithms progress, we anticipate that the transparency of these applications will also enhance. We were also of the opinion that the therapeutic utility of AI might be shown by prospective studies.
